# Thulium fiber laser in cystine calculi

**DOI:** 10.1590/S1677-5538.IBJU.2023.0024

**Published:** 2023-03-31

**Authors:** João Pedro Machado Gismondi, Afonso da Silva Alves Bento, Eduardo Mazzucchi, William Carlos Nahas

**Affiliations:** 1 Universidade de São Paulo Departamento de urologia São Paulo SP Brasil Departamento de urologia, Universidade de São Paulo - USP, São Paulo, SP, Brasil; 2 Universidade de São Paulo Hospital das Clínicas São Paulo SP Brasil Hospital das Clínicas Universidade de São Paulo - USP, São Paulo, SP, Brasil; 3 Instituto do Câncer do Estado de São Paulo Divisão de Urologia São Paulo SP Brasil Divisão de Urologia, Instituto do Câncer do Estado de São Paulo (ICESP) - São Paulo, SP, Brasil

## Abstract

**Introduction:**

Thulium Fiber Laser (TFL) is the most modern technology to treat nephrolithiasis and ureterolithiasis in endourology. Although there are a lot of new studies coming up, we still don't have data on how this laser works in some rare diseases. Cystinuria is the most common genetic nephrolithiasis disorder (1), known for its recurrent lithiasis (2).

Our main goal in this video is to show a successful case of cystine calculi treated with Thulium Fiber Laser (Laser Fiber Dust/Quanta System™). Cystinuria is the most common genetic nephrolithiasis disorder (1), known for its recurrent lithiasis (2).

**Materials and Methods:**

A 25 years-old male, cystinuric, presented with a CT scan, showing a 10mm stone on the right side and two calculi 6 and 7 mm on the left side, all located in the lower calyx. Bilateral flexible ureteroscopy was done using a reusable digital flexible ureteroscope.

Starting on the left side, we repositioned the stone from the lower to the upper calyx, using a tipless front opening basket. Lithotripsy was performed using TFL. Settings were 100 Hz (frequency) and 100 mJ (energy) for dusting. Popcorn technique was also used, setting the laser at 100Hz and 200 mJ, obtaining good dusting. On the right side, lithotripsy was performed in the inferior calyx, also resulting in “snowstorm”.

Procedure time was 120 minutes.

**Results:**

The postoperative was uneventful. Follow up CT showed a 3 mm residual fragment in the right kidney.

**Conclusion:**

This video demonstrates the treatment of bilateral cystine calculi with Thulium Fiber Laser. Reasonable procedure time and excellent dusting results are encouraging, pointing towards great improvements in endourology.
